# A Genome-Wide Association Study Suggests Novel Loci Associated with a Schizophrenia-Related Brain-Based Phenotype

**DOI:** 10.1371/journal.pone.0064872

**Published:** 2013-06-21

**Authors:** Johanna Hass, Esther Walton, Holger Kirsten, Jingyu Liu, Lutz Priebe, Christiane Wolf, Nazanin Karbalai, Randy Gollub, Tonya White, Veit Roessner, Kathrin U. Müller, Tomas Paus, Michael N. Smolka, Gunter Schumann, IMAGEN Consortium, Markus Scholz, Sven Cichon, Vince Calhoun, Stefan Ehrlich

**Affiliations:** 1 Department of Child and Adolescent Psychiatry, University Hospital Carl Gustav Carus, Dresden University of Technology, Dresden, Germany; 2 Institute for Medical Informatics, Statistics and Epidemiology (IMISE), University of Leipzig, Leipzig, Germany; 3 LIFE (Leipzig Interdisciplinary Research Cluster of Genetic Factors, Phenotypes and Environment), University of Leipzig, Leipzig, Germany; 4 Department of Electrical and Computer Engineering, University of New Mexico, Albuquerque, New Mexico, United States of America; 5 The MIND Research Network, Albuquerque, New Mexico, United States of America; 6 Department of Genomics, Life & Brain Center, University of Bonn, Bonn, Germany; 7 Institute of Human Genetics, University of Bonn, Bonn, Germany; 8 Max Planck Institute of Psychiatry, RG Statistical Genetics, Munich, Germany; 9 MGH/MIT/HMS Martinos Center for Biomedical Imaging, Massachusetts General Hospital, Charlestown, Massachusetts, United States of America; 10 Department of Psychiatry, Massachusetts General Hospital, Boston, Massachusetts, United States of America; 11 Department of Psychiatry and the Center for Magnetic Resonance Research, University of Minnesota, Minneapolis, Minnesota, United States of America; 12 Department of Psychiatry, University Hospital Carl Gustav Carus, Dresden University of Technology, Dresden, Germany; 13 Rotman Research Institute, University of Toronto, Toronto, Canada; 14 Brain and Body Centre, University of Nottingham, Nottingsham, United Kingdom; 15 Montreal Neurological Institute, McGill University, Montreal, Quebec, Canada; 16 King's College London, Institute of Psychiatry, London, United Kingdom; 17 Institute of Neuroscience and Medicine (INM-1), Research Center Juelich, Juelich, Germany; Yale University, United States of America

## Abstract

Patients with schizophrenia and their siblings typically show subtle changes of brain structures, such as a reduction of hippocampal volume. Hippocampal volume is heritable, may explain a variety of cognitive symptoms of schizophrenia and is thus considered an intermediate phenotype for this mental illness. The aim of our analyses was to identify single-nucleotide polymorphisms (SNP) related to hippocampal volume without making prior assumptions about possible candidate genes. In this study, we combined genetics, imaging and neuropsychological data obtained from the Mind Clinical Imaging Consortium study of schizophrenia (n = 328). A total of 743,591 SNPs were tested for association with hippocampal volume in a genome-wide association study. Gene expression profiles of human hippocampal tissue were investigated for gene regions of significantly associated SNPs. None of the genetic markers reached genome-wide significance. However, six highly correlated SNPs (rs4808611, rs35686037, rs12982178, rs1042178, rs10406920, rs8170) on chromosome 19p13.11, located within or in close proximity to the genes *NR2F6*, *USHBP1*, and *BABAM1*, as well as four SNPs in three other genomic regions (chromosome 1, 2 and 10) had p-values between 6.75×10^−6^ and 8.3×10^−7^. Using existing data of a very recently published GWAS of hippocampal volume and additional data of a multicentre study in a large cohort of adolescents of European ancestry, we found supporting evidence for our results. Furthermore, allelic differences in rs4808611 and rs8170 were highly associated with differential mRNA expression in the cis-acting region. Associations with memory functioning indicate a possible functional importance of the identified risk variants. Our findings provide new insights into the genetic architecture of a brain structure closely linked to schizophrenia. *In silico* replication, mRNA expression and cognitive data provide additional support for the relevance of our findings. Identification of causal variants and their functional effects may unveil yet unknown players in the neurodevelopment and the pathogenesis of neuropsychiatric disorders.

## Introduction

Despite a number of twin studies indicating high heritability in complex neuropsychiatric disorders such as schizophrenia [1]–[4], the underlying molecular pathways and mechanisms of susceptibility for these disorders remain elusive. A major issue in psychiatric genetics is the lack of replication of putative risk variants [5]–[7]. Possible reasons for this problem might include the previously widely used candidate gene approach, polygenic inheritance, the genetic and the phenotypic heterogeneity of the disorders, and the low reliability and long-term stability of psychiatric diagnoses.

To address the latter, it has been suggested to use intermediate phenotypes instead of diagnosis, because intermediate phenotypes are thought to be more proximal to the underlying substrate of the illness than the varying clinical constructs. Suitable intermediate phenotypes are traits that are reliably measurable, stable, continuously distributed (so called “quantitative traits”), heritable, and disease-associated [8], [9].

In patients with schizophrenia, a reduction of hippocampal volume has been repeatedly demonstrated [10]–[13]. Abnormalities of the structure and function of the hippocampus in schizophrenia have been associated with deficits in memory and executive function [14], suggesting that these structural changes could reflect a central pathophysiological process associated with the disease [11]. Furthermore, sibling and family studies provide evidence for the heritability (40–70%) of this brain structure [15], [16]. Therefore, it is widely acknowledged that hippocampal volume represents a reliable intermediate phenotype for schizophrenia.

With the rapid development of genotyping technology, genome-wide association studies (GWAS) offer the opportunity to identify biological markers and risk genes that are associated with specific phenotypes by scanning the entire genome. Whereas candidate gene approaches rely on prior and possibly ill-defined assumptions about the underlying biological pathways and neurodevelopmental models of disorders or intermediate phenotypes, a GWAS approach is hypothesis-free.

After the identification of new risk genes, it is crucial to elucidate the function of the genetic variants and their potential contribution to the phenotype or illness. The analysis of gene expression profiles may provide insights into the underlying genetic mechanisms influencing a phenotype. This can be achieved by examining the differential allelic expression of gene products in the same region, which provides additional evidence for the functional relevance of the findings [17], [18]. A complementary strategy is to study the relationship of risk variants to cognitive or behavioural measures which are closely linked to the brain-based phenotype [19].

By combining the power of a GWAS with the use of a well-established brain-based intermediate phenotype we aimed to identify relations between genetic polymorphisms and the hippocampal volume of patients with schizophrenia and demographically similar healthy control subjects. We sought replication of our findings using the very recently published data of the Enhancing Neuro Imaging Genetics through Meta-Analysis (ENIGMA) Consortium [20] and data of the IMAGEN study, a large European multicentre genetic-neuroimaging study of reinforcement behavior in adolescence [21]. An additional aim was to determine possible functional mechanisms of the identified genetic associations by analyzing (a) differential allelic expression using gene expression data from human hippocampus tissue and (b) the relation of risk variants to hippocampus-dependent cognitive functioning.

## Materials and Methods

### Participants

The Mind Clinical Imaging Consortium (MCIC) study of schizophrenia [13], [22] obtained baseline structural MRI scans on a total of 328 subjects from four participating sites: Massachusetts General Hospital in Boston (MGH) and the Universities of Iowa (UI), Minnesota (UMN) and New Mexico (UNM). All subjects gave written informed consent prior to study enrolment. The human subjects research committees at each of the four sites (Massachusetts General Hospital in Boston and the Universities of Iowa, Minnesota and New Mexico) approved the study protocol. We confirm that all potential participants who declined to participate or otherwise did not participate were eligible for treatment (if applicable) and were not disadvantaged in any other way by not participating in the study. During the consent process the subjects were asked a series of questions to assure that they understood the nature of the study, that if they chose to participate it was voluntary and that they could stop at any time without affecting their care, and that they understood the risks and benefits of the study. If they stated that they wanted to participate, they were also asked the reason why they chose to participate. If there was any question as to the ability to provide informed consent (i.e., they don't understand the risks or benefits, or they suffer from acute delusions that could significantly impair a patient's judgment) then they were not recruited for the study. In addition, if during the clinical interview it was determined that they lacked the ability to provide informed consent, then they were dropped from the study at that time. The patient group (SZ) consists of subjects with a DSM-IV diagnosis of schizophrenia, established using structured clinical interviews and review of case files by trained clinicians. Healthy controls (HC) were included if they had no history of a medical or Axis I psychiatric diagnosis. All participants were required to be at least 18 years of age and no older than 60 and to be fluent in English. Participants were excluded if they had a history of neurologic disease, or psychiatric disease other than schizophrenia, history of a head injury with loss of consciousness, history of substance abuse or dependence within the past month, severe or disabling medical conditions, contraindication to MR scanning or IQ less than 70 (based on the reading subtest from the WRAT3). The final sample with complete and high-quality structural MRI and genetic data comprised of 126 HC and 115 SZ. For quality assurance procedures see below.

For replication purpose we obtained additional genetic and sMRI data from participants of (I) the ENIGMA network [20] with a discovery sample of N = 7,795 (including 5,775 healthy individuals and 2,020 patients with depression, anxiety, Alzheimer's disease or schizophrenia), and (II) the IMAGEN study [21] containing N = 1,663 healthy 14-year old adolescents (for detailed information see Supporting Information (SI) 1.1. in File S1).

### Clinical Measures

Prior to subject enrolment, clinicians from all four MCIC sites participated in a two-day training session, during which cross-site inter-rater reliability for the primary diagnostic and symptom-rating scales was established (>85% concordance with videotaped training materials). All study participants underwent an extensive clinical diagnostic assessment that included either the SCID-I/P or NP [23] or the Comprehensive Assessment of Symptoms and History (CASH) [24]. Premorbid cognitive achievement was estimated by the Wide Range Achievement Test (WRAT3-RT) [25]; parental socioeconomic status (SES) was determined using the Hollingshead index [26] and handedness was determined using the Annett Scale of Hand Preference [27]. Severity of positive and negative symptoms were rated using the Scale for the Assessment of Positive Symptoms (SAPS) and the Scale for the Assessment of Negative Symptoms (SANS) [28], [29]. Antipsychotic history was collected as part of the psychiatric assessment using the PSYCH instrument [30] and cumulative and current antipsychotic exposure was calculated using the chlorpromazine (CPZ) conversion factors [31]. See Table 1 and Table S1 in File S1 for detailed information.

**Table 1:** Demographic variables of the MCIC sample.

Scanner Fieldstrength	Sample		Sex (female)		Ethnicity (White)		Age (years)		WRAT3-RT		Parental SES		Handedness		Hippocampal Volume	
		N	N	%	N	%	mean	SD	mean	SD	mean	SD	mean	SD	mean	SD
1.5T	HC	107	42^a^	39.3	80	74.8	32.07^b^	10.83	50.96^b^	4.05	2.76	0.71	0.81	2.51	8814.80^b^	859.13
	SCZ	85	22^a^	25.9	55	64.7	35.91^b^	11.10	46.56^b^	7.06	2.92	1.04	1.28	3.34	8318.12^b^	989.96
3T	HC	19	7	36.8	17^a^	89.5	31.89	11.26	51.00^b^	3.94	2.37	0.76	0.47	0.77	8929.00^b^	817.21
	SCZ	30	8	26.7	18^a^	60.0	32.43	10.45	45.97^b^	6.09	2.63	0.85	1.67	3.43	8328.13^b^	849.54
Total	HC	126	49^a^	38.9	97^a^	77.0	32.05^b^	10.85	50.97^b^	4.02	2.70	0.73	0.76	2.33	8832.02^b^	850.75
	SCZ	115	30^a^	26.1	73^a^	63.5	35.00^b^	11.01	46.40^b^	6.79	2.84	0.99	1.38	3.35	8320.73^b^	951.70

Means and standard deviations (SD) are given. HC = healthy control, SZ = patient with schizophrenia. Ethnicity was defined as described under Methods. WRAT3-RT = Wide Range Achievement Test 3 – Reading Test. Parental SES (socioeconomic status) was classified according to Hollingshead, and handedness determined using the Annett Scale of Hand Preference.

a significantly different between HC and SZ on basis of Chi-Square (p<0.05).

b significantly different between HC and SZ on basis of Welch (p<0.05).

### Structural Image Acquisition

MCIC structural MRI data were acquired with either a 1.5T Siemens Sonata (MGH, UI, UNM) or a 3T Siemens Trio (UMN). The T1-weighted structural brain scans at each of the four sites were acquired with a coronal gradient echo sequence: TR = 2530 ms for 3T, TR = 12 ms for 1.5T; TE = 3.79 for 3T, TE = 4.76 ms for 1.5T; TI = 1100 for 3T; Bandwidth = 181 for 3T, Bandwidth = 110 for 1.5T; 0.625×0.625 voxel size; slice thickness 1.5 mm; FOV, 256×256×128 cm matrix; FOV = 16 cm; NEX = 1 for the 3T, NEX = 3 for the 1.5T. Cross site MRI acquisition calibration and reliability were established in a preceding study using human phantoms, following guidelines developed by the biomedical informatics research network (BIRN) test bed for morphometry [32], [33].

### Structural Image Data Processing

MCIC structural MRI data from three consecutive volumes were registered, motion corrected, averaged and analyzed in an automated manner with atlas-based FreeSurfer software suite (http://surfer.nmr.mgh.harvard.edu, Version 4.0.1). This process included volumetric segmentation, cortical surface reconstruction [34]–[37] and the estimation of total intracranial volume (ICV) [38]. Hippocampal volume is a standard output of the FreeSurfer volumetric segmentation [35]. Previous imaging genetics studies have shown the same genetic effects for the left and right hippocampus [39], [40]. Therefore we used mean hippocampal volume (averaged across the right and left hemisphere) as the primary parameter for analysis. Segmentation and surface reconstruction quality were assured by manual inspection of all raw MRI volumes, segmented volumes in three planes and pial as well as inflated volumes. Five participants' MRI data failed the aforementioned quality assurance. The data of these subjects were then recovered with minor manual intervention following the FreeSurfer user guidelines.

### Genotyping

Blood samples were obtained of each MCIC participant and sent to the Harvard Partners Center for Genetics and Genomics. DNA extraction and genotyping was performed according to the manufacturer's protocol and blinded for group assignment (SI 1.2. in File S1). Genotyping was performed at the Mind Research Network (MRN) Neurogenetics Core Lab using the Illumina HumanOmni-Quad BeadChip interrogating 1,140,419 SNPs. Normalized bead intensity data obtained for each sample were loaded into GenomeStudio2010 software, which generated SNP genotypes from fluorescent intensities using the manufacturer's default cluster settings. The raw genotypic data were imported into a genome-wide data management system (Laboratory Information Management System) to allow the tracking of individual samples, quality control and the export of user defined formats compatible with the genetic programs used for statistical analysis.

Quality control steps included a per-individual quality control, i.e. identification and exclusion of individuals with a) discordant sex information, b) missing genotype information of more than 5%, c) unusual heterozygosity rate (details see below), d) divergent ancestry (see paragraph about population stratification below) and e) duplicated or related individuals, and a per-marker quality control (identification and exclusion of SNPs with f) an excessive missing genotype rate of more than 10%, g) significantly different missing genotype rates between cases and controls, and h) a minor allele frequency below 5%) [41], [42]. All steps were carried out in PLINK [43]. For the initial 255 samples, the total genotyping rate was 99.8%. Sex was estimated based on SNP data and was in line with self-disclosure. Due to excess heterozygosity we excluded two control samples (outliers defined as mean heterozygosity +/−4SD). Testing for random (call rate <90%) and non-random missing genotype data (haplotypic case/control test with p<1×10^−10^) led to the exclusion of 657 SNPs. Another 194,543 SNPs were excluded because of a minor allele frequency less than 0.05, resulting in a final dataset of 743,591 autosomal SNPs.

### Statistics

For each of the 743,591 SNPs tested for association in the MCIC sample, we used PLINK [43] to fit a linear regression model with minor allele count, sex, age, diagnosis, ICV and scanner field strength as predictors of total hippocampal volume. We modeled the effects of diagnosis (i.e. healthy individual or participant with schizophrenia) to account for non-random sampling and possible additional environmental factors specific to psychiatric patients such as treatment effects or stress.

As population stratification is a well-known issue in heterogeneous data sets and can become problematic especially in association studies, we needed to correct for allele frequency differences that are due to systematic ancestry differences. We applied principal component analysis (PCA) to our genotype data using EIGENSTRAT of the EIGENSOFT 3.0 software package [44], [45]. Before PCA, SNP data were pruned based on LD as recommended [46]. We also excluded autosomal SNPs, SNPs in problematic regions of long-range linkage disequilibrium (LD) (as recommended by Price et al. [47]), and all SNPs in a +/−500 kb range of SNPs found in the “GWAS Catalog” (http://www.genome.gov/admin/gwascatalog.txt, accessed on 21/6/11) to be possibly associated with hippocampal volume or schizophrenia, resulting in 103,860 SNPs. The first 10 principal components (based on Tracy-Widom-Statistic, see Table S2 in File S1) were used as additional covariates in our regression model (see above).

To verify our results in an ethnically homogeneous sample we defined a subsample based on stringent criteria, including individuals of European descent only. For this purpose, we again performed EIGENSTRAT-based PCA using the pruned SNP set as defined above to analyze our sample in combination with four HapMap populations (CHB = Han Chinese in Beijing, China, JPT = Japanese in Tokyo, Japan, YRI = Yoruba in Ibadan, Nigeria, and CEU = Utah residents with ancestry from northern and western Europe; International HapMap Project http://www.hapmap.org/). Based on this analysis, a homogeneous subsample of individuals close to the CEU cluster was selected, (n = 170; see SI 1.3 in File S1 and Figure S1 for further details).

### Replication Analyses

For replication, we chose all top-ranking SNPs of our MCIC association analysis, i.e. markers with p-values smaller than 10^−5^. We then checked for association signals with bilateral hippocampal volume for the aforementioned SNPs (if available) and all other available intragenic SNPs in a window of +/−100 kb of our top SNPs (I) using EnigmaVis, an online interactive visualization tool of genome-wide association signals of the ENIGMA study [48], and (II) estimating similar linear regression models as described above using the IMAGEN data.

### Differential Allelic Expression in Human Hippocampus

Biopsy samples were obtained from 142 patients with chronic pharmacoresistant temporal lobe epilepsy. After quality control, fresh frozen human hippocampal segments of 138 individuals were prepared as tissue slices under cryostat conditions (Bonn tissue bank) and total DNA and RNA were isolated using AllPrep DNA/RNA Micro Kit (Qiagen, Hilden, Germany). A volume of 50 ng of total RNA was amplified (Illumina TotalPrep 96-RNA Amplification Kit, Ambion/Applied Biosystems, Darmstadt, Germany) and labelled cRNA was hybridised to Illumina human HT-12 Expression v3 BeadChips (Illumina, San Diego, CA, USA). All expression profiles were extracted using GenomeStudio software (Illumina). For genome-wide SNP-genotyping of these individuals, 200 ng of DNA were hybridised to Illumina Human660W-Quad v1 DNA Analysis Bead-Chip (Infinium HD Assay Super manual, Illumina).

The sequences of expression probes were re-aligned to UCSC version 18 (hg18, http://genome.ucsc.edu/) allowing only perfect matches, and then normalized using the vsn2 option implemented in the package ‘VSN’ for R. For quantitative trait analysis, linear regression of an additive allelic model predicting mRNA expression was performed using the GenABEL package for R (http://www.genabel.org/), including the covariates gender, age at sampling, and the first five components resulting from multidimensional scaling analysis of the genotype data carried out in PLINK [43]. For further details see SI 1.4. in File S1 and [17].

### Association with Hippocampus-dependent Cognitive Functioning

To test for possible effects of single putative genetic risk variant (identified in the MCIC sample using the linear regression models described above) on hippocampus-dependent cognitive functioning we applied structural equation modeling (SEM) following the guidelines set forth by Arbuckle and Wothke (1999) using AMOS 18.0 with full maximum likelihood estimation. We hypothesized that the risk polymorphism would have an indirect negative effect on memory functioning, which would be mediated via hippocampal volume. “Memory”, the dependent variable, was designed as a latent variable defined by two different neuropsychological measures tapping hippocampus-dependent memory-functions (see SI 1.5. in File S1) which were available for 198 subjects. For reasons of simplicity, we included only the first two most significant principal components (see above; Table S2 in File S1) to correct the independent variable – the genetic polymorphism – for population stratification. Hippocampal volume (adjusted for the effects of ICV and scanner field strength) was specified as mediator variable and we explicitly modeled the effects of age, sex and diagnosis on hippocampal volume and memory.

## Results

### Sample characteristics

MCIC patients and controls did not differ significantly in parental socioeconomic status or handedness. Patients were slightly older, less likely to be female, included fewer participants of European descent, had lower WRAT3-RT scores and, as expected, a significantly smaller mean hippocampal volume (Table 1). For an overview of the clinical variables of the patient group see Table S1 in File S1. We also found no differences in demographic or clinical variables when stratifying the sample according to the acquisition site-specific scanner field strength.

### GWAS

**Figure 1:**
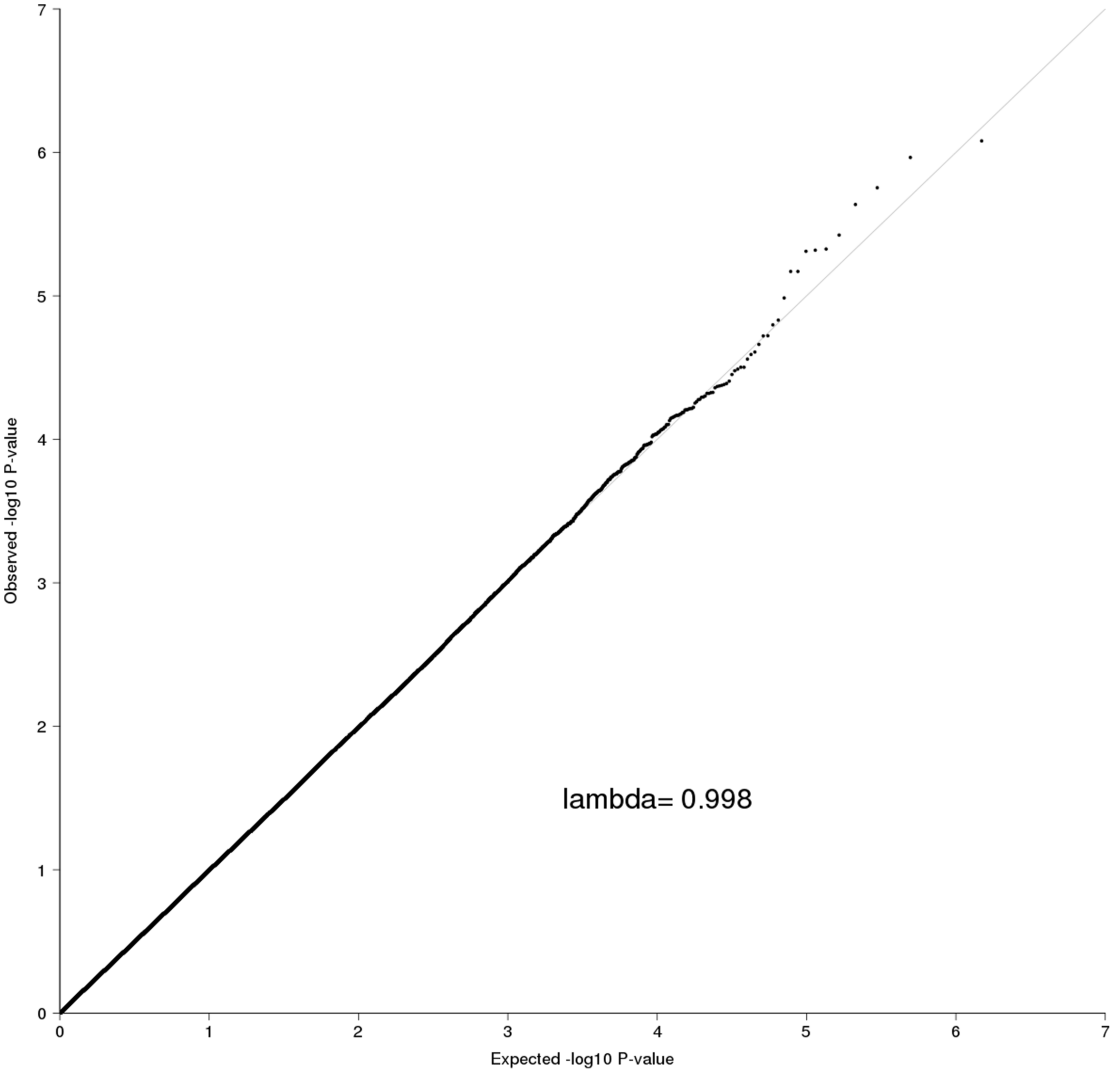
Quantile-quantile plot for MCIC association results. The empirical and theoretical distributions are shown as dots and line, respectively.

We tested each of the 743,591 SNPs in the MCIC sample using multiple linear regression models for association with human hippocampal volume as described above. Figure 1 shows the quantile-quantile (QQ) plot. An inflation factor of λ = 0.998 was estimated, indicating that there is no inflation of false-positive results derived from genotyping errors or uncontrolled population stratification. No marker exceeded the widely acknowledged genome-wide significance threshold of 5×10^−8^ [49].

**Figure 2:**
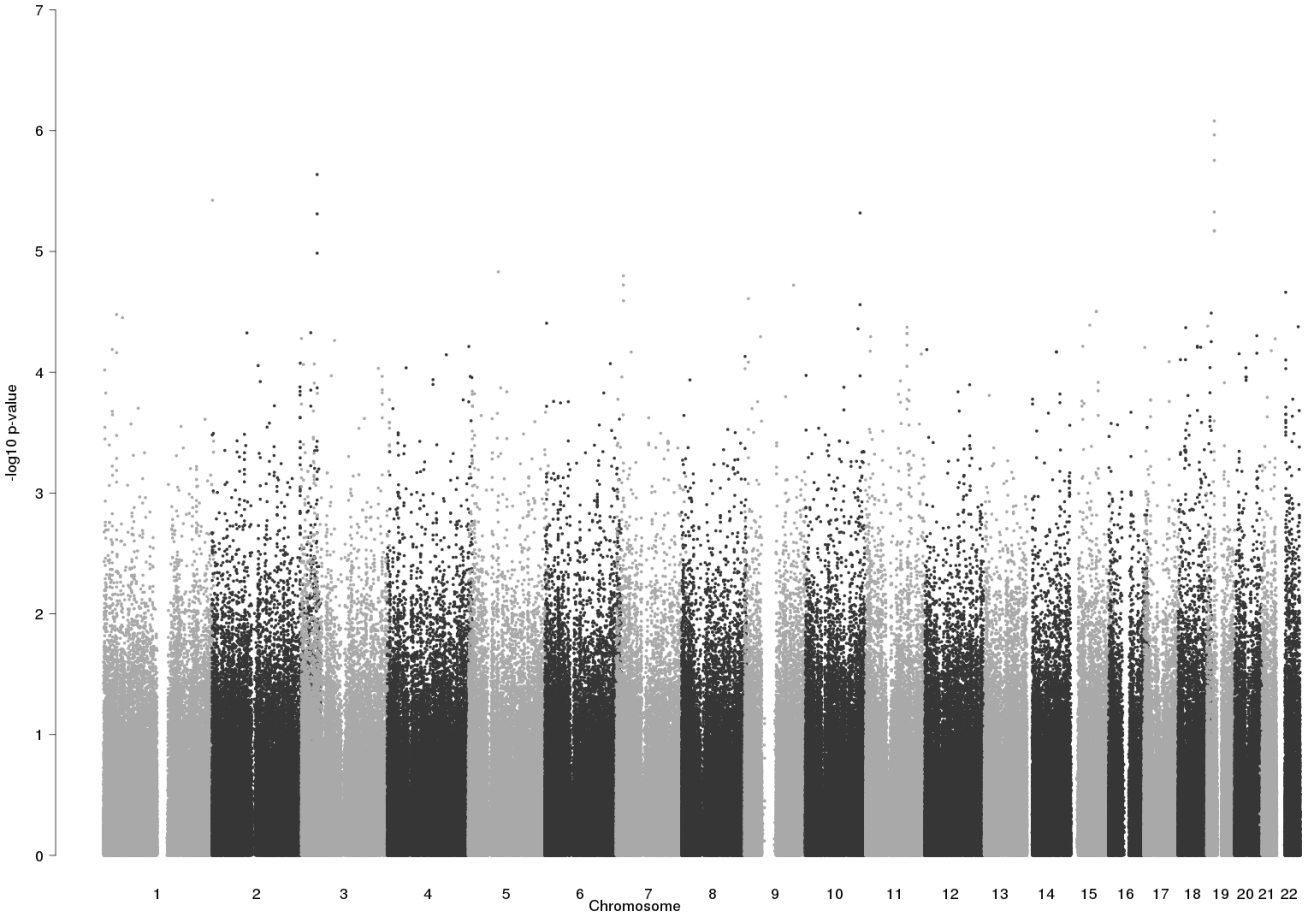
Genome-wide association results of hippocampal volume in the MCIC sample. Negative logarithmic p-values are plotted against their genomic position.

Assuming that the most significantly associated SNPs comprise variants which are actually influencing hippocampal volumes, in the following we focus on the ten loci having p-values smaller than 1×10^−5^. The smallest p-value (p = 8.3×10^−7^) was obtained for SNP rs35686037, which is located 3,384 bases upstream of *NR2F6* (nuclear receptor subfamily 2 group F member 6) and 1,314 bases downstream of *USHBP1* (Usher syndrome 1C binding protein 1) on chromosome 19. Furthermore, we found four associated markers with a p-value smaller than 1×10^−5^ on chromosome 1 (within *KIF26B*), 2 (within or near *TRPM8*) and 10 (*LOC283089*). An overview of the top-SNPs and corresponding gene regions is shown in Table 2; a Manhattan plot of the p-values is shown in Figure 2. Additional information about the distribution of genotypes, call rate, and heterozygosity rates can be found in Table S3 in File S1, regression coefficients, standard errors and corresponding confidence intervals for each of the ten SNPs are given in Table S4 in File S1 and Figure S2.

**Table 2:** Genome-wide association results for SNPs associated with hippocampal volume in the MCIC sample.

SNP	CHR	BP	A1	A2	MAF	BETA	STAT	P	Gene/Region
rs9919234	1	243770613	G	T	0.4046	310.6	4.742	3.766×10^−06^	KIF26B (intron)
rs11901004	2	234591999	T	G	0.1287	−436.6	−4.686	4.885×10^−06^	TRPM8 (UTR 3′)
rs17866592	2	234594425	C	T	0.1354	−446.7	−4.851	2.305×10^−06^	TRPM8 (1,521 bases downstream)
rs1254152	10	122572603	G	A	0.3880	307.9	4.688	4.798×10^−06^	LOC283089 (intron)
rs4808611	19	17215825	T	C	0.1743	391.2	4.692	4.711×10^−06^	NR2F6 (intron)
rs35686037	19	17220535	T	C	0.1680	431.8	5.071	8.305×10^−07^	USHBP1 (1,214 bases downstream)
rs12982178	19	17232568	C	T	0.1896	416.3	5.015	1.083×10^−06^	USHBP1 (intron)
rs10424178	19	17240558	T	C	0.2095	390.8	4.909	1.761×10^−06^	BABAM1 (intron)
rs10406920	19	17250648	T	C	0.1805	376.1	4.610	6.750×10^−06^	BABAM1 (intron)
rs8170	19	17250704	A	G	0.1805	376.1	4.610	6.750×10^−06^	BABAM1 (coding-synon)

SNP IDs with chromosome (CHR), basepair position (BP), minor (A1) and major allele (A2), minor allele frequency (MAF), regression coefficient (BETA), coefficient (STAT) and asymptotic p-value for t-statistic, and corresponding gene regions: *KIF26B* (kinesin family member 26B), *TRPM8* (transient receptor potential cation channel, subfamily M, member 8), *LOC283089* (uncharacterized), *NR2F6* (nuclear receptor subfamily 2, group F, member 6), *USHBP1* (Usher syndrome 1C binding protein 1), and *BABAM1* (BRISC and BRCA1 A complex member 1). For additional information see Table S3 in File S1.

There were no significant pairwise relationships between the ten gene loci and either sex, ethnicity, age, WRAT3-RT scores, parental SES or handedness (data not shown). We also inspected the results for association with left and right hippocampal volume separately. Additionally, we tested for association with hippocampal volume in a model without covarying for diagnostic status (Table S5 in File S1) and in a homogeneous subsample of individuals with ancestry from north-western Europe (defined as described above; see also SI 1.3. in File S1). As can be seen in Table S5 in File S1 and in Table 3, all ten loci showed again significant effects with p-values smaller than 5.5×10^−4^ and the direction of the effects was the same. Furthermore, we tested for association with hippocampal volume in a subsample of only patients and only healthy controls, respectively. All ten SNPs exceeded nominal significance in each group and again, the direction of these effects were the same (Table 3).

**Table 3:** P-values of 10 MCIC major findings in subanalyses.

SNP	CHR	LeftHippoVol	RightHippoVol	Subset of European descent	Group of SZ patients	Healthy controls
rs9919234	1	1.705×10^−06^	5.425×10^−05^	5.011×10^−05^	1.572×10^−03^	5.848×10^−04^
rs11901004	2	4.055×10^−05^	5.126×10^−06^	5.551×10^−04^	5.115×10^−04^	5.780×10^−03^
rs17866592	2	1.146×10^−05^	4.761×10^−06^	4.727×10^−04^	2.560×10^−04^	3.614×10^−03^
rs1254152	10	8.934×10^−06^	2.101×10^−05^	3.835×10^−04^	1.743×10^−04^	5.290×10^−03^
rs4808611	19	7.865×10^−06^	2.274×10^−05^	3.315×10^−04^	2.216×10^−02^	6.033×10^−06^
rs35686037	19	1.370×10^−06^	5.589×10^−06^	7.826×10^−05^	1.381×10^−02^	1.155×10^−06^
rs12982178	19	2.847×10^−06^	4.763×10^−06^	7.515×10^−05^	3.943×10^−02^	1.477×10^−07^
rs10424178	19	1.688×10^−06^	1.618×10^−05^	3.319×10^−05^	2.893×10^−02^	1.676×10^−06^
rs10406920	19	7.452×10^−06^	4.321×10^−05^	3.740×10^−04^	1.434×10^−02^	4.289×10^−05^
rs8170	19	7.452×10^−06^	4.321×10^−05^	3.740×10^−04^	1.434×10^−02^	4.289×10^−05^

Association with left and right hippocampal volume and association with hippocampal volume in a MCIC subsample of European descent was analyzed controlling for the same variables as in our main GWAS models. Association of hippocampal volume in a group of only patients with schizophrenia (N = 115) or only healthy controls (N = 126) was controlled for gender, scanner field strength differences, age, and ICV.

**Figure 3:**
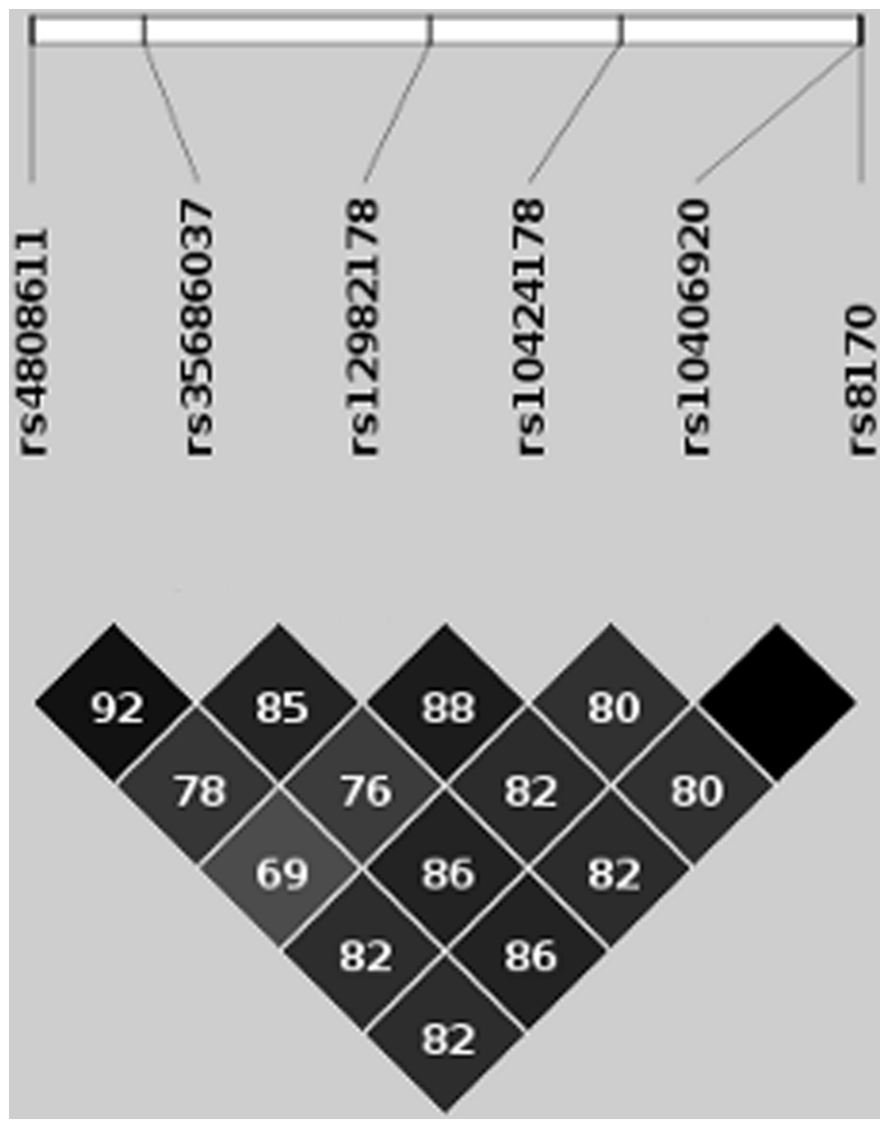
Linkage disequilibrium (LD) plot of all MCIC main hits on chromosome 19. LD is given based on r^2^ estimated using the current dataset. Each diamond indicates the pairwise magnitude of LD, with dark grey/black indicating strong LD (r^2^>0.8). Figure prepared with *HaploView* (Barrett et al. 2005).

The strongest evidence for association in our main analysis as well as in subsequent analyses (see above) was found for six highly correlated SNPs (rs4808611, rs35686037, rs12982178, rs10424178, rs10406920, and rs8170) on chromosome 19. The LD structure of these six markers is shown in Figure 3. The genomic region, characterized by high LD, includes three genes: *NR2F6*, *USHBP1*, and *BABAM1* (*BRISC and BRAC1 A complex member 1*; also referred to as *C19orf62*). Rs8170 is the only SNP (of all SNPs with p<1×10^−5^) located in a coding region (coding-synonymous K (AAG)→K (AAA)).

For the most significant SNP in this LD block (rs35686037) we found a reduction in hippocampal volume of approximately 5% per risk allele (∼391 mm^3^ compared to the mean hippocampal volume of 8588 mm^3^). This corresponds to an effect size of Cohen's f^2^ = 0.115 and an explained variance of 5.72% (calculated as explained variance in addition to the variance explained by the control variables in the linear model).

Using the *in silico* replication strategy outlined above, we found the following significant association signals in the genomic neighborhood (+−100 kb) of our main hits: In close proximity of rs9919234 on chromosome 1 we found 17 SNPs associated with hippocampal volume (e.g. rs1472051 with p = 8.7×10^−3^) in the ENIGMA sample, and two SNPs in the IMAGEN sample, all belonging to the same gene (*KIF26B*). On chromosome 2 we found 20 associated SNPs (e.g. rs763379 with p = 9.2×10^−3^; 2 kb upstream of rs17866592) in the ENIGMA sample, and three other SNPs (e.g. rs617970 with p = 3.2×10^−4^) in the IMAGEN sample. Each of these SNPs is located in the same or in the adjacent gene (*TRPM8*, *SPP2*) or the intergenic region between those two genes. Close to rs1254152 on chromosome 10 we identified 31 SNPs in association with hippocampal volume (e.g. rs7911084 with p = 1.4×10^−3^) in the ENIGMA sample, and rs12570141 with p = 2.9×10^−2^ in the IMAGEN sample. For the interconnected genomic region on chromosome 19 we searched a wider window (300 kb) and found 12 associated SNPs (e.g. rs4808629 with p = 3.5×10^−3^) in the ENIGMA sample, and 12 further SNPs (e.g. rs2278897 with p = 5.1×10^−4^) in the IMAGEN sample, all close to our top-ranking SNPs rs480811, rs35686087 or rs8170. An overview of all relevant SNPs is given in Table S6 and S7 in File S1.

### Differential Allelic Expression in Human Hippocampus

Only five of our ten main findings were part of the differential allelic expression analysis in human hippocampus tissue (rs9919234, rs17866592, rs1254152, rs4808611, rs8170). However, based on LD (Figure 3) the latter two SNPs were identified to function as the most relevant proxies for the missing SNPs on chromosome 19 and rs17866592 can serve as proxy for rs11901004 on chromosome 2 (r^2^ = 1). In a cis-region of the six markers of chromosome 19 (defined using a window of +−1 mega basepairs (Mb)) we identified the minor alleles of rs4808611 and rs8170 (as well as of rs35686037, rs12982178, rs10424178 and rs10406920 based on LD) to be highly associated with lower expression of *ABHD8* and *MRPL34* (p = 2.7×10^−5^ and p = 7.6×10^−5^, respectively; *Bonferroni*-corrected for the number of transcripts in the cis-region). Both genes are in head-to-head orientation to each other and located ca. 0.12 Mb downstream of BABAM1.

### Association with Hippocampus-dependent Cognitive Functioning

In order to explore possible indirect effects of risk polymorphisms on memory functioning we compared different structural equation models: Model 1 did neither include SNP nor hippocampus effects on memory, Model 2 comprised direct SNP effects on memory but no effects of hippocampus and finally Model 3 included direct effects of SNP on hippocampus as well as direct effects of hippocampus on memory (Figure S3). The comparison of established model fit indices [50] and information criteria [51], [52] revealed Model 3 as the best fit (Table S6 in File S1). In this model the negative effect of risk alleles on memory functioning are mediated by hippocampal volume. The size and direction of all effects are depicted in Figure S3 for SNP rs35686037, while the negative indirect effects of each of the six genetic risk variants of chromosome 19 are listed in Table S8 in File S1.

## Discussion

By performing genome-wide association analyses of an intermediate phenotype, we identified novel genetic loci that are associated with hippocampal volume, as measured by MRI in patients with schizophrenia and in healthy controls. Six highly correlated SNPs in a LD block on chromosome 19p13.11 and four SNPs in three genomic regions on chromosome 1, 2 and 10 showed p-values between 6.7×10^−6^ and 8.3×10^−7^ in the GWA models. The SNPs on chromosome 19 were strongly associated with altered gene expression in human hippocampus tissue. Furthermore, our *in silico* replication analysis, using large datasets of the ENIGMA study and IMAGEN studies, provides supporting evidence for our association results.

Due to the clustering of our findings in the chromosome 19 region (providing additional support for the validity of these findings), we will first discuss these six SNPs. The genes corresponding to the aforementioned SNPs on chromosome 19p13.11 are protein-coding and feature a direct or indirect association with hippocampus and brain development. *NR2F6* is an orphan nuclear receptor also known as *EAR2*. It has been shown to influence DNA binding, ligand-dependent nuclear receptor activity, zinc ion binding, sequence specific DNA binding transcription factor activity and hormone receptor activity [53], [54]. Furthermore, *NR2F6* is involved in neural development, signal transduction and as a co-regulator of thyroid hormone nuclear receptor and glucocorticoid receptor functioning [55]. The latter function involves physical and functional interactions with *NR3C1*, a glucocorticoid receptor, which plays a major role in regulation of the hypothalamic-pituitary-adrenocortical (HPA) system. Glucocorticoids exert negative feedback control on the HPA axis by regulating hippocampal and paraventricular nucleus neurons [56]. Oversecretion of glucocorticoids caused by sustained stress can damage the feedback response and cause hippocampal atrophy [57], [58]. Genetic variants in *NR3C1* variants contribute to the genetic programming of the individual's set point of HPA axis activity and may be involved in the deregulation of HPA axis activity by biological or psychosocial stress, trauma, and early life experiences [56]. Accordingly, *NR3C1* variants have been associated with hippocampal volume and unipolar depression [59].

*BABAM1* plays a role in DNA repair and chromatin modification [60] and *USHBP1* interacts via its C-terminus with the first PDZ domain of the Usher syndrome 1C protein, which is coded by one of several genes responsible for the Usher syndrome - a relatively rare genetic disorder that is a leading cause of deafness and gradual blindness [61]. These genes have important functions in the development and stability of the cell layers of the retina. The retina is a part of the central nervous system (and often used as a model in developmental brain cell culture studies) and it may thus be speculated whether genetic variants in *USHBP1* are associated with developmental abnormalities in the arrangement of neurons in cell layers in other brain regions, such as the hippocampus, as well.

According to the Allen Brain Atlas (Allen Institute for Brain Science; http://human.brain-map.org/) all three genes (*NR2F6*, *USHBP1*, *BABAM1*) in the highly associated LD block on chromosome 19 are expressed in human brain. As an example, the expression of NR2F6 in human hippocampus is shown in Figure S4. Furthermore, we could show that SNPs in the aforementioned genes influence the expression of proximal genes in human resected hippocampi in an allele-wise manner. The newly identified risk variants in *NR2F6*, *USHBP1*, and *BABAM1* are associated with the expression of *ABHD8* (abhydrolase domain containing 8), important for hydrolase activity [62], and *MRPL34* (mitochondrial ribosomal protein L34), a structural constituent of ribosomes and relevant in translation processes [63]. Our findings of differential allelic expression underline the importance of the identified loci for the expression of genes related to protein synthesis and thus could provide a functional understanding of our genetic association results.

The identified polymorphisms and the corresponding genes *NR2F6*, *USHBP1* and *BABAM1* have not previously been associated with schizophrenia or other neuropsychiatric disorders. Given that our imaging genetics approach is very different from comparing genotypes across cases and controls this is not surprising. However, since hippocampal volume is a well acknowledged intermediate phenotype for schizophrenia our results open up new avenues for psychiatric research. The fact that the effect of the identified genetic variants on hippocampal volumes was not limited to, or greater in, patients with schizophrenia is in line with the intermediate phenotype hypothesis. Using intermediate phenotypes allows for the identification of risk alleles in individuals who do not carry a diagnosis (i.e. healthy controls, siblings or individuals with subthreshold symptoms) assuming that the liability to schizophrenia is stochastic rather than categorical. However, our study cannot answer the question whether the association between the identified SNPs and hippocampal volume is specific to schizophrenia but it has been suggested that a variety of other polymorphisms with small effect sizes, reciprocal effects with risk alleles of other genes, copy number variants and environmental influences may constitute a background of risk factors that could interact with the effects of *NR2F6*, *USHBP1* and *BABAM1* to increase schizophrenia susceptibility. This susceptibility may manifest itself, in part, as a structural change in the medial temporal lobe [64]–[66].

Follow up studies should not only replicate our findings but also relate the identified variants to cognitive or functional markers relevant to neuropsychiatric disorders. We attempted to take a first step into this direction by relating the polymorphisms in the *NR2F6*, *USHBP1*, and *BABAM1* genes - although not genome-wide significant - to hippocampus-dependent cognitive functions, most importantly, verbal and logical memory [67]–[69]. Indeed, our exploratory structural equation models provide additional evidence for an association between the risk SNPs in these genes and impaired memory functioning which was mediated by reduced hippocampal volumes (see Table S6 and Figure S3 in File S1). In support of our findings, histopathological studies have indicated a causal relationship between verbal memory impairments and hippocampal neuron loss in CA3 and the hilar area for patients with left temporal seizure foci [70], [71].

The four remaining SNPs rs9919234, rs11901004, rs17866592, and rs1254152 (see Table 2) belong to *KIF26B* (1q44), *TRPM8* (2q37.1) and an uncharacterized gene region (LOC283089, 10q26.13), respectively. Intervals in 1q44 have been described as critical regions containing genes leading to structural abnormalities of the corpus callosum [72]. The transient receptor potential (*TRP*) superfamily comprises a group of non-selective cation channels that sense and respond to changes in their local environments. In the central nervous system, TRPs participate in neurite outgrowth, receptor signalling and excitotoxic cell death resulting from anoxia [73]. Accordingly, *TRPM8* was found to be a susceptibility loci for common migraine and has been the focus of neuropathic pain models [74].

Previous imaging genetics studies on hippocampal atrophy using a genome-wide approach have all focused on Alzheimer's disease. All three studies [40], [75], [76] are largely based on the same sample obtained via the multicenter Alzheimer's Disease Neuroimaging Initiative [77]. Potkin et al. (2009) used hippocampal grey matter density as intermediate phenotype and identified susceptibility genes for Alzheimer's disease by analyzing interaction effects. Shen et al. (2010) included a variety of imaging phenotypes (grey matter density and volumetric measures) but did not replicate the initial findings. It was concluded, that different imaging phenotypes (i.e. regions and grey matter density vs. volumes) may not be equally sensitive to the same genetic markers and consequently provide complementary information. Finally Furney et al. (2011) found a disease-specific effect of *ZNF292* on entorhinal cortex volume which reached genome-wide significance. Our study design was different from these reports in that we focussed on hippocampal volume and used an independent sample of patients with schizophrenia and healthy controls.

Very recently the ENIGMA consortium published a genome-wide association analysis for mean bilateral hippocampal, total brain and intracranial volume [20]. In a large discovery sample no markers reached genome-wide significance and previously identified polymorphisms associated with hippocampal volume or schizophrenia showed no or little association. The strongest association signal for hippocampal volume after controlling for intracranial volume was reported for two SNPs in the same LD block (rs7294919 and rs7315280 with p = 4.43×10^−7^ and p = 2.42×10^−7^, respectively), located between *HRK* and *FBXW8* (12q24.22). Neither SNP reached genome-wide significance in our sample (p = 0.05565 and p = 0.007548, respectively) or in any of the other studies on the genetics of hippocampal volume described above. Possible reasons for the different main results of the ENIGMA study compared to our own GWAS results comprise the different study design and cohorts (i.e. ENIGMA combined 17 European cohorts, some of them multicentre studies, and the data was obtained using different MRI scanner and MRI data analysis technologies as well as different genotyping platforms across the acquisition sites) as well as a somewhat different statistical approach (i.e. their models included other covariates).

Although the main association signals of the ENIGMA and our own study do not correspond, our *in silico* replication approach using data from the ENIGMA study provides supportive evidence for the validity of our own association signals. Similarly the regression models using genetic and hippocampal volume data of the IMAGEN study revealed SNPs in close proximity to our main hits which were associated with hippocampal volume. Given that the IMAGEN study includes solely 14-year olds, these results indicate that the identified genes or gene regions might exert their influence on hippocampal volume during development.

Nevertheless, the findings of our study have to be considered in the light of the following limitations. Firstly, none of the identified risk variants did reach the commonly accepted genome-wide significance threshold. Our sample set was limited by the number of individuals with genetic information but the use of a quantitative trait design has been shown to substantially increase the statistical power [78]. The fact that six SNPs in strong LD exhibited near-threshold association is encouraging and suggests that our findings were not likely due to genotyping artifact, although the effects may be small. Additionally, validation in a more homogeneous subsample of European descent as well as the gene expression and cognitive functioning analysis lend further support to the relevance of the identified loci. Secondly, the replication data sets did not include equally large numbers of schizophrenia patients (none in the IMAGEN sample) which makes it difficult to compare results and also precludes answering the question whether our findings are specific to schizophrenia. Thirdly, the differential allelic expression analysis was carried out using tissue of patients with chronic pharmacoresistent temporal lobe epilepsy. Although possibly more reliable than using post mortem brain tissue, epilepsy may affect non-coding DNA regulatory elements in some cells in a different way than schizophrenia or at-risk states for schizophrenia [79]. Finally, although the pattern of our results seems to point to developmental mechanisms, the hippocampus is subject to a variety of environmental influences such as physical exercise or stress effects mediated by the HPA [80], [81]. Such effects could either blur earlier developmental effects or they could themselves be moderated by genetic polymorphisms or epigenetic mechanisms [82]. To disentangle these complicated relationships, gene-environment interaction studies are warranted – unfortunately our study did not include any measures of stress or cortisol levels.

## Conclusions

Taken together, our findings support previous reports demonstrating that GWAS with a quantitative brain-based intermediate phenotype as a dependent variable is a viable method to identify associated gene variants without making prior assumptions about the underlying biology of the phenotype. Our results were supported by gene expression, cognitive data and similar association signals in the replication samples. Elucidating the specific mechanisms of *NR2F6*, *USHBP1* and *BABAM1* in the regulation of neurodevelopment and synaptic (re)organization could improve our conceptual framework of processes related to hippocampal volume reduction and facilitate a better understanding of schizophrenia. Ultimately, imaging genetics could contribute to the development of methods for earlier detection and tailored therapeutic intervention in schizophrenia and other neuropsychiatric disorders [83].

## Supporting Information

File S1 **SI 1 Material and Methods, SI 2 Results.**
(DOCX)

Figure S1 **First two principal components (PC) plotted against each other for MCIC data and the four HapMap populations.** Definition of European subsample as described above (zoomed in on the right picture). Blue cross marks the center of all HapMap CEU individuals (International HapMap Project http://www.hapmap.org/). Blue circles are the single (inner circle) and 1.5 times (outer circle) the Euclidean distance between this center and the HapMap CEU individual farthest away from this center.
(TIF)

Figure S2 **Forest plot of regression coefficients and corresponding 95% confidence intervals for main hits in the patient group and in healthy controls, respectively (MCIC sample).** SZ = patients with schizophrenia; HC = healthy controls.
(TIF)

Figure S3 **Path model predicting indirect effects of SNP on memory.** Standardized path coefficients are given exemplarily for SNP rs35686037. Indirect effect of marker rs35686037 on “Memory” = −0.047 in the MCIC sample. For all other values see Table S4 in File S1. E1 to e5 are error terms.
(TIF)

Figure S4 **Expression of NR2F6 in human hippocampus.** Hippocampal formation spatially shown in green. Red diamonds represent loci of higher expression compared to other tissues. Figure prepared with *Allen Human Brain Atlas – Brain Explorer 2 (Version 2.2 Build 2312)* of the Allen Institute for Brain Science (Lau et al., 2008).
(TIF)
