# Supplementary material for: A Genome-Wide Association Study Suggests Novel Loci Associated with a Schizophrenia-Related Brain-Based Phenotype
Source: PLoS One. 2013 Jun 21;8(6):e64872. doi: 10.1371/journal.pone.0064872 (PMC3689744; doi:10.1371/journal.pone.0064872)
Supplement: File S1 — SI 1 Material and Methods, SI 2 Results. (DOCX) [file pone.0064872.s001.docx]

**Supporting Information**

**SI 1 Material and Methods**

SI 1.1. Replication samples: Participants, structural imaging data and genotyping

(I) ENIGMA: The ENIGMA discovery sample comprised 17 cohorts of European ancestry from whom genome-wide SNPs and structural MRI data were collected. Unselected population samples and case-control studies were included, with cases ascertained for neuro-psychiatric disorders including depression, anxiety, Alzheimer’s disease and schizophrenia. Protocols for imaging analysis are publicly available on the ENIGMA Consortium website (<http://enigma.loni.ucla.edu/protocols/>). The two most commonly used segmentation packages for sMRI analysis were the FMRIB Software Library (FSL) package of tools and FreeSurfer. For *in silico* replication analysis we used EnigmaVis, an online interactive visualization tool of genome-wide association signals of the ENIGMA study [1]. For more detailed information about the genotyping and the underlying statistical models and different covariates see [2] and the online methods of the ENIGMA consortium (http://enigma.loni.ucla.edu).

(II) IMAGEN: For the present study, 818 adolescent males and 845 females with complete genetic and sMRI data were included. Genome-wide genotyping of ~600,000 autosomal SNPs was performed using the Illumina Quad 610 chips (Illumina, San Diego, CA, USA). Structural MRI was performed on 3T scanners from different manufacturers. Scanning parameters were chosen to be compatible with all scanners [3]. T1-weighted images were acquired from each participant using a modified protocol based on the ADNI project (http://adni.loni.ucla.edu/research/protocols/mri-protocols/). The images comprised 160 slices with 1.1 x 1.1 x 1.1 mm^3^ voxel size. Preprocessing, i.e. segmentation and volume labeling was performed using the fully automated FreeSurfer pipeline (Version 5.0). Gender, acquisition site and intracranial volume were additional covariates for the linear regression models to test for possible association between a subset of SNPs (main findings of our GWAS and flanking SNPs) and hippocampal volume.

SI 1.2. Genotyping of the MCIC sample

Approximately 200 ng of DNA were used to genotype each subject sample according to the manufacturer’s protocol (Illumina HumanOmni-Quad BeadChip, Illumina, San Diego, CA). After amplification, fragmentation and hybridization, the specifically hybridized DNA was fluorescently labeled by a single base extension reaction and detected using an iScan scanner. Non-specifically hybridized fragments were removed by washing, while remaining specifically hybridized DNA were processed for the single base extension reaction, stained and imaged on an Illumina iScan Reader.

SI 1.3. MCIC Subsample of European descent

Focusing on the first two principal components (see Figure S1) we defined European origin as having a distance no larger than 1.5 times the Euclidean distance between the center of all HapMap CEU individuals and the HapMap CEU individual farthest away from this center. We then excluded all subjects without measures for their hippocampal volume. In order to further ensure homogeneity of this subsample, we performed a second EIGENSTRAT-based PCA on the 172 remaining subjects. Here we excluded outliers based on the 6SD default criterion as defined in EIGENSTRAT's standard settings.

SI 1.4. Differential Allelic Expression in Human Hippocampus

Quality of total RNA of all samples was checked for degradation (RNA integrity number RIN47.9) via BioAnalyzer measurements (Agilent Technologies, Waldbronn, Germany).

Individuals were deﬁned as outliers and excluded from analysis if their coordinates on at least one of the ﬁrst two axes of the multi-dimensional scaling analysis on the identical by state matrix of genotyped SNPs was more than six standard deviations away from the mean position of all other individuals. Quality thresholds were as follows: HWE p-value ≥ 1×10^-5^, MAF ≥ 1%, individual callrate ≥ 98%, SNP callrate ≥ 98%, and a false discovery rate of 1% for autosomal heterozygosity. Average genotyping rate was 98%.

SI 1.5. Neuropsychological Measures

The cognitive assessments were conducted by psychometrists and were supervised by experienced neuropsychologists who had participated in an in person standardization training for the four sites (for details see [4]). For the current analysis we chose 2 scales representative of key cognitive functions which have been associated with the hippocampus and are affected in patients with schizophrenia: Verbal learning was measured using the Hopkins Verbal Learning Test-Revised [5]. Logical Memory was assessed using a subtest (recall for verbally presented stories) of the Wechsler Memory Scale-III [6].

**Table S1: Clinical variables of patients with schizophrenia.**

Means and standard deviations (SD) are given. We found no differences in length of illness, positive and negative symptoms or cumulative lifetime antipsychotic medication across the acquisition sites with different scanner fieldstrength.

| **Scanner Fieldstrength** | **Sample Size** | **Length of Illnes** [years] | | **Positive Symptoms** | | **Negative Symptoms** | | **Medication** [cum. dose/year] | |
| --- | --- | --- | --- | --- | --- | --- | --- | --- | --- |
|  |  | mean | SD | mean | SD | mean | SD | mean | SD |
| 1.5T | 85 | 12.82 | 11.00 | 4.82 | 2.93 | 8.34 | 4.12 | 57.27 | 122.32 |
| 3T | 30 | 9.76 | 8.79 | 4.93 | 2.38 | 7.43 | 2.92 | 44.16 | 100.71 |
| Total | 115 | 12.01 | 10.52 | 4.85 | 2.79 | 8.10 | 3.85 | 53.90 | 116.86 |

**Table S2: Principal component statistics from the MCIC Eigenstrat analysis.**

The program uses Tracy-Widom (TW) statistics to test if the means of the eigenvector coordinates associated to each individual across population differ significantly.

| **Eigenvector** | **Eigenvalue** | **TW statistic** | **TW p-value** |
| --- | --- | --- | --- |
| 1 | 9.675 | 273.864 | < 1 × 10^-50^ |
| 2 | 3.382 | 363.213 | < 1 × 10^-50^ |
| 3 | 1.609 | 177.270 | < 1 × 10^-50^ |
| 4 | 1.190 | 28.928 | 5.461 × 10^-47^ |
| 5 | 1.188 | 29.730 | 7.027 × 10^-49^ |
| 6 | 1.179 | 27.822 | 1.989 × 10^-44^ |
| 7 | 1.174 | 27.533 | 9.123 × 10^-44^ |
| 8 | 1.168 | 26.921 | 2.245 × 10^-42^ |
| 9 | 1.166 | 27.659 | 4.700 × 10^-44^ |
| 10 | 1.162 | 27.622 | 5.725 × 10^-44^ |

**SI 2 Results**

**Table S3: Distribution of genotypes, call rate and heterozygosity rate for main hits in the MCIC sample.**

Distribution of genotypes (GENO) is given in the following order: homozygous in minor allele/heterozygous/homozygous in major allele (see Table 2 for minor allele frequency). CR = call rate; HET = heterozygosity.

| **SNP ID** | **CHR** | **BP** | **GENO** | **CR** | **HET** |
| --- | --- | --- | --- | --- | --- |
| rs9919234 | 1 | 243770613 | 39/117/85 | 1.000 | 0.4855 |
| rs11901004 | 2 | 234591999 | 5/51/181 | 0.983 | 0.2152 |
| rs17866592 | 2 | 234594425 | 5/55/180 | 0.996 | 0.2292 |
| rs1254152 | 10 | 122572603 | 36/115/90 | 1.000 | 0.4772 |
| rs4808611 | 19 | 17215825 | 8/68/165 | 1.000 | 0.2822 |
| rs35686037 | 19 | 17220535 | 7/67/167 | 1.000 | 0.2780 |
| rs12982178 | 19 | 17232568 | 8/75/157 | 0.996 | 0.3125 |
| rs10424178 | 19 | 17240558 | 11/79/151 | 1.000 | 0.3278 |
| rs10406920 | 19 | 17250648 | 10/67/164 | 1.000 | 0.2780 |
| rs8170 | 19 | 17250704 | 10/67/164 | 1.000 | 0.2780 |

**Table S4: Regression coefficients, standard errors and confidence intervals for main hits in the patient group and in healthy controls, respectively (MCIC sample).**

Multiple linear regression models were used to test for association with human hippocampal volume in the group of patients with schizophrenia (SZ) and in healthy controls (HC). Regression coefficients (BETA), standard errors (SE) and 95% confidence intervals (L95 = lower range; U95 = upper range) are given for each of the main hits.

|  | | **SZ** | | | | **HC** | | | |
| --- | --- | --- | --- | --- | --- | --- | --- | --- | --- |
| **SNP ID** | **CHR** | **BETA** | **SE** | **L95** | **U95** | **BETA** | **SE** | **L95** | **U95** |
| rs9919234 | 1 | 353.8 | 109.1 | 140.0 | 567.7 | 282.6 | 80.0 | 125.8 | 439.4 |
| rs11901004 | 2 | -541.7 | 151.2 | -838.0 | -245.5 | -320.9 | 114.1 | -544.6 | -97.2 |
| rs17866592 | 2 | -555.8 | 147.0 | -843.9 | -267.6 | -337.1 | 113.5 | -559.6 | -114.6 |
| rs1254152 | 10 | 391.5 | 100.7 | 194.1 | 588.8 | 237.3 | 83.5 | 73.6 | 400.9 |
| rs4808611 | 19 | 303.0 | 130.6 | 47.1 | 558.9 | 479.4 | 101.2 | 281.0 | 677.7 |
| rs35686037 | 19 | 331.8 | 132.6 | 71.9 | 591.6 | 532.0 | 103.8 | 328.6 | 735.4 |
| rs12982178 | 19 | 272.1 | 130.5 | 16.3 | 527.9 | 548.0 | 98.0 | 355.8 | 740.2 |
| rs10424178 | 19 | 281.1 | 127.0 | 32.2 | 529.9 | 472.9 | 93.8 | 289.1 | 656.8 |
| rs10406920 | 19 | 326.7 | 131.3 | 69.4 | 584.0 | 411.0 | 96.7 | 221.4 | 600.5 |
| rs8170 | 19 | 326.7 | 131.3 | 69.4 | 584.0 | 411.0 | 96.7 | 221.4 | 600.5 |

**Table S5: P-values of the regression coefficients for the effect of SNP for our main findings from a linear model including all covariates (full model) and another linear model including all covariates except diagnostic status (w/o Dx).**

SNP = single nucleotide polymorphism; CHR = chromosome number.

| **SNP** | **CHR** | **P** | **P** (w/o Dx) |
| --- | --- | --- | --- |
| rs9919234 | 1 | 1.705 × 10^-06^ | 8.168 × 10^-06^ |
| rs11901004 | 2 | 4.055 × 10^-05^ | 8.267 × 10^-06^ |
| rs17866592 | 2 | 1.146 × 10^-05^ | 4.391 × 10^-06^ |
| rs1254152 | 10 | 8.934 × 10^-06^ | 9.956 × 10^-07^ |
| rs4808611 | 19 | 7.865 × 10^-06^ | 2.006 × 10^-05^ |
| rs35686037 | 19 | 1.370 × 10^-06^ | 5.776 × 10^-06^ |
| rs12982178 | 19 | 2.847 × 10^-06^ | 1.689 × 10^-05^ |
| rs10424178 | 19 | 1.688 × 10^-06^ | 1.579 × 10^-05^ |
| rs10406920 | 19 | 7.452 × 10^-06^ | 2.566 × 10^-05^ |
| rs8170 | 19 | 7.452 × 10^-06^ | 2.566 × 10^-05^ |

**Table S6: P-values for MCIC main hits and flanking SNPs in the ENIGMA sample.**

All SNPs with p < 0.05 are displayed with their genomic position and their effect (meta-analytic effect size), standard error and p-value as found using the EnigmaVis tool available online using hippocampal volume as the dependent variable (controlling for population stratification, age, age^2^, sex and the interactions between age and sex as well as age^2^ and sex, intracranial volume, and dummy covariates for different scanner sequences or equipment within a site when needed). The SNP with the smallest p-value in each gene region is displayed in bold. CHR = chromosome; BP = base pair position; SE = standard error.

| **SNP ID** | **CHR** | **BP** | **Effect** | **SE** | **p-value** |
| --- | --- | --- | --- | --- | --- |
| *Genomic region for rs9919234* | | | | | |
| rs1069232 | 1 | 243641337 | -13.5686 | 6.1799 | 0.02812 |
| rs12564551 | 1 | 243678513 | -14.1787 | 6.7581 | 0.0359 |
| rs12094249 | 1 | 243679519 | -13.0353 | 6.3458 | 0.03996 |
| **rs1472051** | **1** | **243692333** | **-16.7117** | **6.3685** | **0.008687** |
| rs1173657 | 1 | 243692662 | 14.2493 | 6.1747 | 0.02102 |
| rs1173660 | 1 | 243700890 | -21.3412 | 9.6802 | 0.02748 |
| rs1538471 | 1 | 243708842 | 13.3052 | 6.2853 | 0.03427 |
| rs1538469 | 1 | 243718502 | -114.89 | 53.6368 | 0.03219 |
| rs11588538 | 1 | 243747503 | 15.9046 | 7.5893 | 0.03611 |
| rs1771503 | 1 | 243781895 | -50.1021 | 24.0725 | 0.03741 |
| rs1771499 | 1 | 243784757 | -62.5066 | 30.5446 | 0.04072 |
| rs6428936 | 1 | 243808393 | 23.779 | 11.1792 | 0.03341 |
| rs6699248 | 1 | 243810042 | -24.2123 | 11.2794 | 0.03183 |
| rs7513390 | 1 | 243812150 | 23.6267 | 11.1532 | 0.03414 |
| rs1771522 | 1 | 243845607 | 13.5945 | 6.0876 | 0.02554 |
| rs10802227 | 1 | 243851917 | -14.5964 | 6.9867 | 0.03669 |
| rs10754459 | 1 | 243852131 | -14.9435 | 7.0076 | 0.03297 |
| *Genomic region for rs17866592* | | | | | |
| rs10490014 | 2 | 234537251 | 18.5722 | 8.1798 | 0.02318 |
| rs12185625 | 2 | 234540172 | -19.6755 | 8.4265 | 0.01955 |
| rs12995095 | 2 | 234541110 | 20.1961 | 9.1006 | 0.02647 |
| rs6709823 | 2 | 234541494 | 22.3378 | 8.6336 | 0.009673 |
| rs6760498 | 2 | 234543000 | 23.7301 | 9.1175 | 0.009249 |
| **rs763379** | **2** | **234592520** | **-48.5859** | **18.6499** | **0.009183** |
| rs17865708 | 2 | 234632149 | 26.1294 | 11.8467 | 0.02741 |
| rs11562947 | 2 | 234633253 | -27.0387 | 11.5007 | 0.01872 |
| rs17869099 | 2 | 234633893 | 23.4367 | 11.8697 | 0.04833 |
| rs4663351 | 2 | 234642717 | 23.1221 | 11.7003 | 0.04813 |
| rs6716659 | 2 | 234643397 | 35.5237 | 16.6985 | 0.03339 |
| rs11563027 | 2 | 234644909 | -33.3964 | 16.515 | 0.04316 |
| rs6719734 | 2 | 234649550 | 35.4334 | 16.5581 | 0.03236 |
| rs10929333 | 2 | 234655592 | -27.8901 | 12.9638 | 0.03145 |
| rs16850380 | 2 | 234666980 | -14.7906 | 6.6665 | 0.02651 |
| rs250938 | 2 | 234667561 | 11.4501 | 5.714 | 0.04508 |
| rs250933 | 2 | 234672230 | -16.8777 | 7.0444 | 0.01658 |
| rs250931 | 2 | 234672627 | -11.8222 | 5.7699 | 0.04047 |
| rs250927 | 2 | 234674529 | 11.7828 | 5.8737 | 0.04485 |
| rs13395103 | 2 | 234679154 | -23.5083 | 11.958 | 0.04931 |
| *Genomic region for rs1254152* | | | | | |
| rs11199515 | 10 | 122475799 | -14.2676 | 5.1163 | 0.01588 |
| rs9421389 | 10 | 122489690 | 13.0408 | 5.6092 | 0.02008 |
| rs17584106 | 10 | 122510144 | -29.9992 | 11.4631 | 0.00887 |
| rs12764375 | 10 | 122534194 | -21.1449 | 9.3628 | 0.02392 |
| rs7092824 | 10 | 122553883 | 17.4389 | 7.2141 | 0.01563 |
| rs7071661 | 10 | 122559974 | -13.103 | 6.4748 | 0.043 |
| rs9919401 | 10 | 122560805 | -14.2203 | 5.6827 | 0.01234 |
| rs7901805 | 10 | 122561412 | -15.4609 | 5.7118 | 0.006792 |
| rs1439465 | 10 | 122574436 | 21.8842 | 9.3927 | 0.01981 |
| rs2997227 | 10 | 122575842 | 16.4195 | 6.3699 | 0.009947 |
| rs11199598 | 10 | 122600647 | 14.7193 | 5.9955 | 0.01409 |
| rs11199602 | 10 | 122606885 | 13.8748 | 5.9344 | 0.01939 |
| rs10886788 | 10 | 122610241 | -13.7327 | 5.9656 | 0.02134 |
| rs11199604 | 10 | 122610606 | 14.6455 | 5.9522 | 0.01387 |
| rs3758507 | 10 | 122612828 | 14.2608 | 5.9568 | 0.01666 |
| rs10886789 | 10 | 122614669 | -14.5859 | 5.9523 | 0.01427 |
| rs4751800 | 10 | 122626720 | 22.9378 | 8.533 | 0.007185 |
| rs3758509 | 10 | 122628850 | -14.747 | 5.9854 | 0.01375 |
| rs3758510 | 10 | 122629160 | 14.6564 | 5.9752 | 0.01417 |
| rs4372362 | 10 | 122634982 | 14.4967 | 5.9874 | 0.01547 |
| rs7079527 | 10 | 122636103 | -19.1441 | 6.4695 | 0.003085 |
| rs754869 | 10 | 122645708 | -19.3653 | 6.4927 | 0.002858 |
| rs12415091 | 10 | 122646609 | -19.4514 | 6.2793 | 0.00195 |
| rs10788126 | 10 | 122647189 | 16.3542 | 5.9361 | 0.005868 |
| rs2289336 | 10 | 122649480 | -19.2965 | 6.2958 | 0.002177 |
| **rs7911084** | **10** | **122649958** | **-19.8193** | **6.2103** | **0.001416** |
| rs1866518 | 10 | 122651094 | 19.6302 | 6.1956 | 0.001533 |
| rs1652727 | 10 | 122653575 | -16.8405 | 6.0961 | 0.005736 |
| rs1530116 | 10 | 122657012 | 19.27 | 6.1376 | 0.001692 |
| rs7077126 | 10 | 122658286 | -19.129 | 6.0992 | 0.001711 |
| rs1045179 | 10 | 122658791 | 17.7852 | 6.007 | 0.003069 |
| *Genomic region for rs35686037* | | | | | |
| rs4808628 | 19 | 17348620 | -36.0401 | 14.6681 | 0.01401 |
| rs7257450 | 19 | 17349607 | -16.4287 | 7.5738 | 0.03007 |
| rs10403836 | 19 | 17350328 | -23.0646 | 11.4542 | 0.04405 |
| rs3815904 | 19 | 17363895 | -16.8613 | 7.4745 | 0.02408 |
| **rs4808629** | **19** | **17364996** | **-25.845** | **8.839** | **0.003456** |
| rs2303683 | 19 | 17374715 | 37.1935 | 15.0405 | 0.0134 |
| rs12972417 | 19 | 17386314 | 15.2733 | 7.5894 | 0.04417 |
| rs2288408 | 19 | 17395693 | 52.0738 | 22.9497 | 0.02327 |
| rs890840 | 19 | 17402182 | 55.0898 | 26.5301 | 0.03785 |
| rs1035872 | 19 | 17407855 | -21.5364 | 8.1864 | 0.008519 |
| rs11666579 | 19 | 17451281 | 16.5166 | 6.35 | 0.009295 |
| rs11672333 | 19 | 17469516 | -13.6134 | 6.811 | 0.04564 |

**Table S7: P-values for MCIC main hits and flanking SNPs in the IMAGEN sample.**

All SNPs with p < 0.05 are displayed (in PLINK output format) with their genomic position and their regression coefficient (BETA), t-statistic coefficient (STAT) and p-value. The SNP with the smallest p-value in each gene region is bolded, the MCIC SNPs are italicized. CHR = chromosome; BP = base pair position.

| **SNP** | **CHR** | **BP** | **BETA** | **STAT** | **p-value** |
| --- | --- | --- | --- | --- | --- |
| *Genomic region for rs9919234* | | | | | |
| **rs1715794** | **1** | **243834835** | **-44.29** | **-1.988** | **0.04701** |
| rs12031183 | 1 | 243966324 | 46.24 | 1.968 | 0.04922 |
| *Genomic region for rs17866592* | | | | | |
| rs7557532 | 2 | 234397803 | -94.22 | -2.064 | 0.03915 |
| rs502059 | 2 | 234402914 | 45.04 | 1.996 | 0.04608 |
| **rs617970** | **2** | **234627728** | **-124.1** | **-3.607** | **0.0003189** |
| *Genomic region for rs1254152* | | | | | |
| **rs12570141** | **10** | **122438883** | **84.48** | **2.185** | **0.02905** |
| *Genomic region for rs35686037* | | | | | |
| **rs2278997** | **19** | **17027981** | **85.77** | **3.481** | **0.0005129** |
| rs12459084 | 19 | 17031885 | 55.76 | 2.464 | 0.01385 |
| rs7254154 | 19 | 17039119 | 60.15 | 2.663 | 0.00781 |
| rs1979260 | 19 | 17054551 | 45.43 | 2.074 | 0.03821 |
| rs7246865 | 19 | 17080105 | 60.91 | 2.445 | 0.0146 |
| rs3826689 | 19 | 17159893 | -50.21 | -2.246 | 0.02483 |
| rs962917 | 19 | 17163247 | -50.49 | -2.223 | 0.02638 |
| rs1545620 | 19 | 17164774 | -50.45 | -2.246 | 0.02482 |
| rs1064305 | 19 | 17166440 | -49.64 | -2.204 | 0.02765 |
| rs8108998 | 19 | 17175874 | -52.57 | -2.336 | 0.0196 |
| rs12463169 | 19 | 17182669 | -52.58 | -2.337 | 0.01958 |
| rs8106139 | 19 | 17367176 | 63.2 | 2.018 | 0.04379 |

**Table S8: Size and direction of indirect SNP effects on “Memory”**

For each of the six reported SNPs on chromosome (CHR) 19 we found a negative indirect effect on “Memory” in the MCIC sample. Neuropsychological measures are described above (SI 1.4.). Χ^2^ statistics, Akaike information criterion (AIC) [7], Browne-Cudeck criterion (BCC) [8] and the goodness-of fit index (GFI) [9] are given for Model 1 - 3. Standardized indirect effects are given for Model 3.

| **SNP ID** | **CHR** | **BP** | **Model 1** | | | | **Model 2** | | | | **Model 3** | | | | **SNP effect on “Memory”** |
| --- | --- | --- | --- | --- | --- | --- | --- | --- | --- | --- | --- | --- | --- | --- | --- |
|  |  |  | **Χ^2^** (df=42) | **AIC** | **BCC** | **GFI** | **X^2^** (df=41) | **AIC** | **BCC** | **GFI** | **X^2^** (df=41) | **AIC** | **BCC** | **GFI** |  |
| rs4808611 | 19 | 17215825 | 181.098 | 229.098 | 232.212 | 0.875 | 179.756 | 229.756 | 232.999 | 0.876 | 177.236 | 227.236 | 230.480 | 0.879 | -0.041 |
| rs35686037 | 19 | 17220535 | 185.904 | 233.904 | 237.017 | 0.873 | 183.723 | 233.723 | 236.966 | 0.874 | 182.042 | 232.042 | 235.285 | 0.876 | -0.047 |
| rs12982178 | 19 | 17232568 | 189.285 | 237.285 | 240.398 | 0.872 | 186.242 | 236.242 | 239.486 | 0.873 | 185.423 | 235.423 | 238.666 | 0.874 | -0.050 |
| rs10424178 | 19 | 17240558 | 189.726 | 237.726 | 240.839 | 0.871 | 186.962 | 236.962 | 240.205 | 0.873 | 185.864 | 235.864 | 239.107 | 0.874 | -0.045 |
| rs10406920 | 19 | 17250648 | 186.417 | 234.417 | 237.530 | 0.872 | 183.420 | 233.420 | 236.664 | 0.874 | 182.555 | 232.555 | 235.798 | 0.876 | -0.041 |
| rs8170 | 19 | 17250704 | 186.417 | 234.417 | 237.530 | 0.872 | 183.420 | 233.420 | 236.664 | 0.874 | 182.555 | 232.555 | 235.798 | 0.876 | -0.041 |

**References**

1. Novak NM, Stein JL, Medland SE, Hibar DP, Thompson PM, et al. (2012) EnigmaVis: online interactive visualization of genome-wide association studies of the Enhancing NeuroImaging Genetics through Meta-Analysis (ENIGMA) consortium. Twin Res Hum Genet 15: 414–418. doi:10.1017/thg.2012.17.

2. Stein JL, Medland SE, Vasquez AA, Hibar DP, Senstad RE, et al. (2012) Identification of common variants associated with human hippocampal and intracranial volumes. Nat Genet 44: 552–561. doi:10.1038/ng.2250.

3. Schumann G, Loth E, Banaschewski T, Barbot A, Barker G, et al. (2010) The IMAGEN study: reinforcement-related behaviour in normal brain function and psychopathology. Mol Psychiatry 15: 1128–1139. doi:10.1038/mp.2010.4.

4. Sponheim SR, Jung RE, Seidman LJ, Mesholam-Gately RI, Manoach DS, et al. (2010) Cognitive deficits in recent-onset and chronic schizophrenia. J Psychiatr Res 44: 421–428. doi:10.1016/j.jpsychires.2009.09.010.

5. Brandt J (1991) The Hopkins Verbal Learning Test: development of a new memory test with six equivalent forms. The Clinical Neuropsychologist 5: 125–142.

6. Wechsler D (1997) Wechsler Memory Scale. 3rd ed. San Antonio, TX: Psychological Corporation.

7. Akaike H (1973) Information theory and an extension of the maximum likelihood principle. Proc 2nd Inter Symposium of Information Theory: 267–281.

8. Browne MW, Cudeck R (1989) Single sample cross-validation indices for covariance structures. Multivariate Behavioral Research: 445–455.

9. Jöreskog KG, Sörbom D (1984) LISREL VI analysis of linear structural relationships by maximum likelihood, instrumental variables, and least square methods. University of Uppsala, Dept of Statistics.

10. Lau C, Ng L, Thompson C, Pathak S, Kuan L, et al. (2008) Exploration and visualization of gene expression with neuroanatomy in the adult mouse brain. BMC Bioinformatics 9: 153. doi:10.1186/1471-2105-9-153.
