# Supplementary figures and images for: A Genome-Wide Association Study Suggests Novel Loci Associated with a Schizophrenia-Related Brain-Based Phenotype
Source: PLoS One. 2013 Jun 21;8(6):e64872. doi: 10.1371/journal.pone.0064872 (PMC3689744; doi:10.1371/journal.pone.0064872)

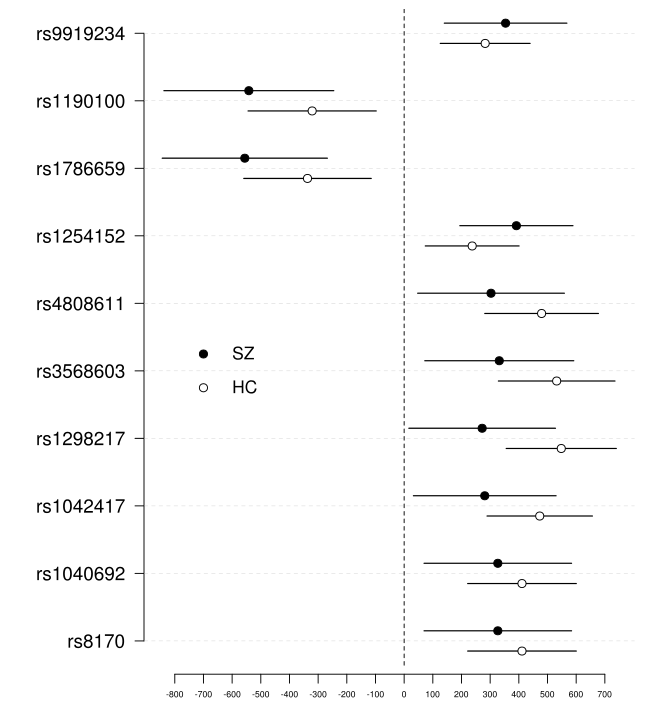

Supplement: Figure S2 — Forest plot of regression coefficients and corresponding 95% confidence intervals for main hits in the patient group and in healthy controls, respectively (MCIC sample). SZ = patients with schizophrenia; HC = healthy controls. (TIF) [file pone.0064872.s003.tif]

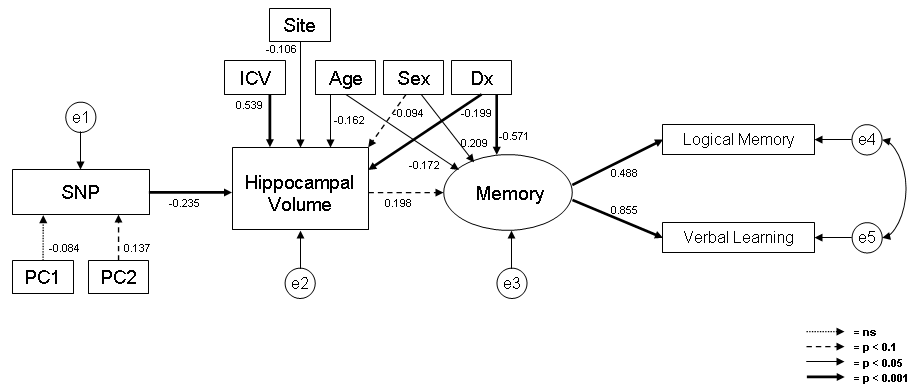

Supplement: Figure S3 — Path model predicting indirect effects of SNP on memory. Standardized path coefficients are given exemplarily for SNP rs35686037. Indirect effect of marker rs35686037 on “Memory” = −0.047 in the MCIC sample. For all other values see Table S4 in File S1. E1 to e5 are error terms. (TIF) [file pone.0064872.s004.tif]

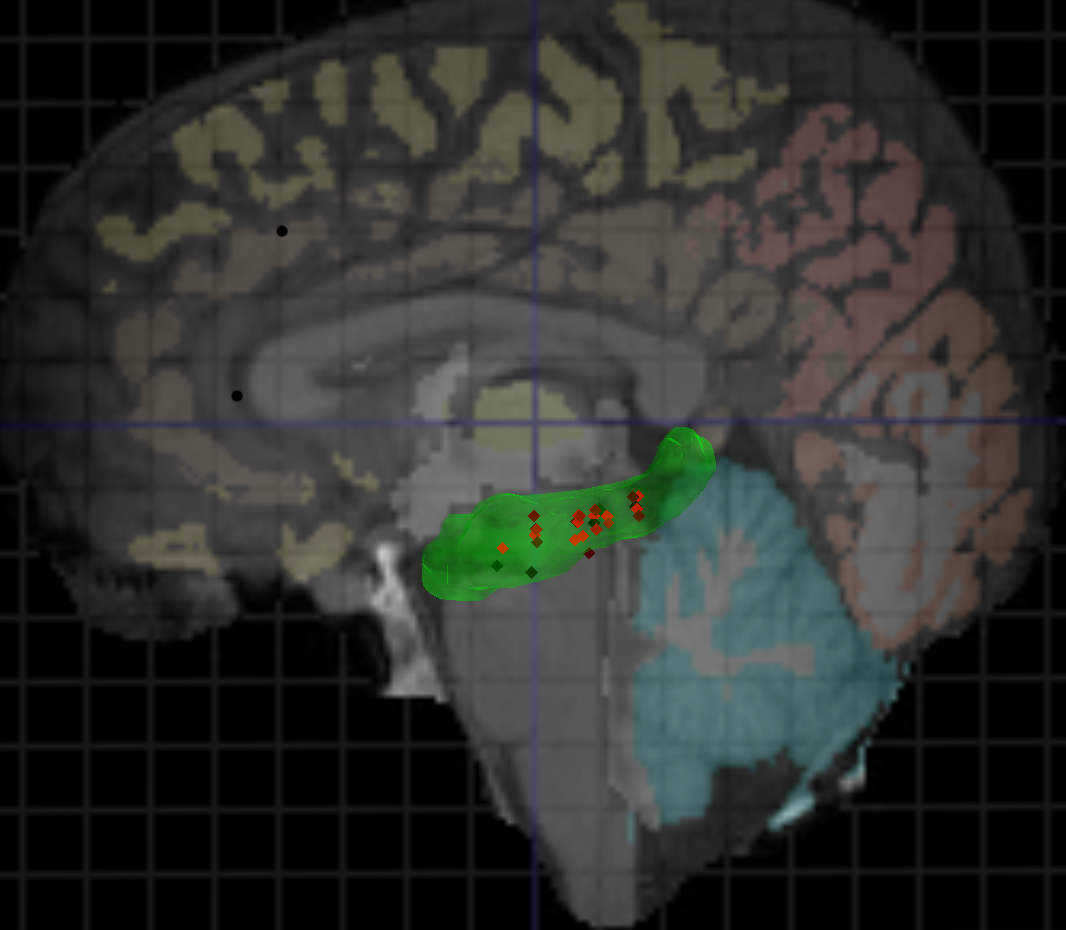

Supplement: Figure S4 — Expression of NR2F6 in human hippocampus. Hippocampal formation spatially shown in green. Red diamonds represent loci of higher expression compared to other tissues. Figure prepared with Allen Human Brain Atlas – Brain Explorer 2 (Version 2.2 Build 2312) of the Allen Institute for Brain Science (Lau et al., 2008). (TIF) [file pone.0064872.s005.tif]
